# Profil socio-démographique et économique des adultes drépanocytaires régulièrement suivis au Centre Hospitalier Universitaire de Libreville

**DOI:** 10.11604/pamj.2022.41.294.28686

**Published:** 2022-04-12

**Authors:** Marielle Igala, Graziella Dolorès Helley Ondo, Léonie Esther Ledaga Lentombo, Léonard Kouegnigan Rerambiah, Stéphane Diop Lacombe, Josaphat Iba Ba, Jean Bruno Boguikouma

**Affiliations:** 1 Service de Médecine Interne, Centre Hospitalier Universitaire de Libreville, Libreville, Gabon,; 2 Département de Médecine Interne et Spécialités Médicales, Université des Sciences de la Santé, Libreville, Gabon,; 3 Laboratoire d´Hématologie et de Biochimie, Centre Hospitalier Universitaire de Libreville, Libreville, Gabon,; 4 Institut de Cancérologie d´Akanda, Akanda, Gabon

**Keywords:** Drépanocytose, socio-démographique, économique, suivi régulier, Sickle cell disease, socio-demographic, economic, regular monitoring

## Abstract

La drépanocytose est une maladie génétique héréditaire de transmission autosomique récessive. L´organisation depuis septembre 2016 au Centre Hospitalier Universitaire de Libreville (CHUL), d´une consultation d´hématologie dédiée à l´adulte drépanocytaire, a été l´occasion de mener cette étude dont le but était de décrire le profil socio-démographique et économique des patients régulièrement suivis. Il s´agissait d´une étude rétrospective, descriptive et non comparative, qui s´est déroulée de septembre 2016 à juin 2019 inclus au service de Médecine Interne du CHUL. Les patients drépanocytaires homozygotes de 18 ans et plus, de tous les genres, suivis durant la période de l´étude, capable de répondre aux questions et qui avaient effectué au moins trois consultations d´hématologie ont été inclus. Un total de 88 patients répondait aux critères d´inclusion sur les 233 vus durant la période de l´étude. Les femmes étaient les plus nombreuses. Le sex ratio était de 0,5 et l’âge moyen de 30,4 ± 7,8 ans, 42% avaient un niveau d´étude supérieur, 88,6% avaient une assurance maladie qui permettait à 31,8% d´entre eux de se prendre en charge en plus de leurs revenus mensuels. La drépanocytose était un obstacle dans la vie quotidienne et professionnelle des patients qui trouvaient un soutien dans leur famille et chez leurs amis. Cette étude a permis de montrer que les patients drépanocytaires qui adhèrent au suivi sont intégrés dans la société. Ils ont pour la plupart un niveau d´instruction qui leur permet de comprendre leur maladie et une assurance santé qui constitue une aide pour leur prise en charge.

## Abstract (en)

Sickle cell disease is a genetic disease transmitted as an autosomal recessive trait. Since September 2016, at the University Hospital Center of Libreville (UHCL) hematology consultation dedicated to adult patients with sickle cell disease was offered. This was the occasion to conduct this study, the purpose of which was to describe the socio-demographic and economic profile of patients followed up on a regular basis. We conducted a retrospective, descriptive and non-comparative study in the Department of Internal Medicine at the UHCL, from September 2016 to June 2019. Patients aged 18 years and older with homozygous sickle cell disease who had been followed up during the study period, were able to answer questions and had undergone at least three haematology consultations were included in the study. A total of 88 patients out of 233 met the inclusion criteria during the study period; women predominated. The sex ratio was 0.5 and the average age of patients was 30.4 ± 7.8 years, 42% had a higher education level, 88.6% had health insurance that allowed 31.8% of them to take care of themselves, in addition to their monthly income. Sickle cell disease was an obstacle in patients daily and professional lives but they got support from family and friends. This study shows that patients with sickle cell disease who attend follow-up visits are integrated into society. Most of them have an education level that allow them to understand their illness and a health insurance which can help them manage their illness.

## Introduction

La drépanocytose, également appelée hémoglobinose S, sicklémie, ou anémie à cellules falciformes, sickle cell disease (SCD) est une maladie génétique héréditaire de transmission autosomique récessive. Elle résulte d´une mutation ponctuelle du sixième codon du gène Î^2^ globine qui provoque la synthèse d´une hémoglobine anormale (HbS) [1]. Il s´agit de la maladie génétique le plus répandue dans le monde. Environ 7% de la population mondiale est porteuse d´un gène anormal de globine [2]. En Afrique centrale, un nouveau-né sur 30 est drépanocytaire. Au Gabon, les porteurs du gène de la drépanocytose représentent 25% de la population, c´est-à-dire un gabonais sur quatre [3]. La majorité des enfants atteints survivent désormais au-delà de 18 ans grâce à l´amélioration de la prise en charge. La disponibilité de la prophylaxie chez l´enfant, les vaccinations et les thérapies telles que l'hydroxyurée ont contribué à l´amélioration de cette survie [4]. Les jeunes adultes sont vulnérables à la perte de soins lors de la transition de la prestation pédiatrique à celle des adultes. Cette transition survient à un âge où ils sont plus susceptibles d'avoir des complications chroniques de la maladie [5]. Au Gabon, l´absence de système transitionnel entre la consultation pédiatrique et adulte, oblige les adultes drépanocytaires à prolonger leur suivi en pédiatrie au-delà de l´âge requis ou à compter sur le service des urgences pour leurs soins. Les conséquences observées sont nombreuses, tant sur le vécu de la drépanocytose et son impact sur le quotidien de l´adulte, que sur les connaissances scientifiques de la maladie chez cette frange de la population. La mise en place depuis septembre 2016 au Centre Hospitalier Universitaire de Libreville (CHUL) d´une consultation d´hématologie dédiée à l´adulte drépanocytaire, a été l´occasion de mener cette étude, la première réalisée dans le pays, dont le but principal était d´évaluer le profil socio-démographique et économique des patients régulièrement suivis.

## Méthodes

**Cadre de l´étude et participants:** il s´agissait d´une étude rétrospective, à visée analytique, descriptive et non comparative, qui s´est déroulée de septembre 2016 à juin 2019 au service de Médecine Interne du CHUL. Après obtention d´un consentement verbal, tous les patients âgés de 18 ans et plus, qui avaient un diagnostic de drépanocytose homozygote, capables de répondre aux questions et qui avaient effectué au moins trois consultations d´hématologie sur une année ont été inclus. Une fiche de renseignements était adressée aux patients, suivis d´entretiens téléphoniques et du dépouillement des dossiers médicaux.

**Variables:** les facteurs sociodémographiques regroupaient l´âge (exprimés en années), le sexe, le niveau d´études, l´activité professionnelle, la situation matrimoniale, l´assurance santé, la gestion de la maladie au quotidien (prises médicamenteuses, soins traditionnels, religion, soutien familial). Les couts des différents examens biologiques et radiologiques ont été listés, ainsi que les coûts d´hospitalisation (avec et sans assurance), le revenu mensuel des patients ayant un emploi a également été précisé. Les coûts estimés ont été calculés à partir des tarifs pharmaceutiques et paracliniques affichés au Centre Hospitalier Universitaire. Les examens réalisés par les patients au CHUL au moins une fois par an ont été classés en: examens hématologiques: la numération formule sanguine et réticulocytes, la vitesse de sédimentation, le frottis sanguin; examens biochimiques: Protéine C réactive (CRP), transaminase, bilirubine totale et directe, lactico-déshydrogénase (LDH), glycémie, urée, créatinine, ionogramme sanguin; examens sérologiques: sérologie rétrovirale, hépatite virale B et C; examens radiologiques: radiographie du thorax, échographie cardiaque et abdominale.

Les médicaments couramment utilisés comprenaient: le paracétamol, acide folique, hydroxyurée, amoxicilline-acide clavulanique et les céphalosporines de 3^e^ génération surtout lors des hospitalisations. Le Salaire Minimum Interprofessionnel Garanti (SMIG) au Gabon est depuis 2006 évalué à 80 000FCFA (122 euros) avec un Revenu Mensuel Minimum (RMM) à 150 000FCFA (229 euros). Les coûts directs non médicaux (transport, aide à domicile) et les coûts indirects concernant la perte de productivité n´ont pas été évalués.

**Méthodes statistiques:** l´analyse des données a été faite avec le logiciel Epi-Info, version 7.2.3.1. Les variables qualitatives étaient exprimées en pourcentage.

## Résultats

Au total 88 dossiers de drépanocytaires répondaient aux critères d´inclusion sur les 233 vus en consultation durant la période d´étude. Les femmes prédominaient avec un ratio homme/femme de 0,5. L´âge moyen était de 30,4 ± 7,8 ans avec des extrêmes de 18 et 53 ans. La tranche d´âge des 25-29 ans était la plus représentée et regroupait 27,3% des patients. La situation matrimoniale des patients révélait, 3 (3,4%) drépanocytaires étaient mariés, 14 (15,9%) avaient un concubin(e) et la grande majorité des patients, 71/88 (80,7%) étaient célibataires. Parmi ces derniers, 45/71 (63,4%) vivaient avec au moins un parent, 15 (21,2%) avec un membre de leur fratrie ou de leur famille, 6 (8,5%) avec un de leurs enfants et 5 (7%) vivaient seuls.

L´évaluation du niveau d´instruction révélait que 37 (42%) drépanocytaires avaient un niveau d´études supérieures. Seuls 36 (40,9%) avaient une activité professionnelle effectuée à temps plein (Tableau 1). Leurs revenus mensuels excédaient les 200 000FCFA pour 16 d´entre eux et 500 000FCFA pour 5. Ces revenus permettaient à 28 (31,8%) des drépanocytaires de prendre en charge leurs soins de santé en association avec une assurance maladie contractée par 77 soit 88,6% des patients (Tableau 2, Tableau 3). Le coût total des examens a été évalué à 223.125 FCFA sans assurance et 51.025FCFA quand l´assurance couvre 80% des frais. Le coût total des médicaments couramment utilisés ainsi que les forfaits d´hospitalisation, de la consultation et du suivi régulier sans et avec la prise en charge à 80% par les assurances sont repris dans le Tableau 4.

**Tableau 1:** répartition des patients selon les caractéristiques socioprofessionnelles

	Effectif	Fréquence (%)
**Niveau d´études**		
Primaire	7	8,0
Collège	21	23,9
Lycée	23	26,1
Etudes supérieures	37	42,0
Total	88	100,0
**Activité professionnelle**		
Oui	36	40,9
Non	52	59,1
Total	88	100,0
**Profession**		
Temps partiel	12	33,3
Plein temps	24	66,7
Total	36	100,0

**Tableau 2:** répartition des patients selon le revenu, la prise en charge et la couverture sociale

	Effectif	Fréquence (%)
**Revenu mensuel**		
Oui	36	40,9
Non	52	59,1
Total	88	100,0
Niveau de revenu FCFA*		
< 200 000	15	41,7
200 000 - 500 000	16	44,4
> 500 000	5	13,9
Total	36	100,0
**Prise en charge des soins**		
Famille	44	50,0
Patient	28	31,8
Les deux	16	18,2
Total	88	100,0
**Assurance**		
Publique	78	88,6
Privée	2	2,3
Les deux	3	3,4
Aucune	5	5,7
Total	88	100,0

* Taux de change 1 Franc CFA= 0,0015 Euro

**Tableau 3:** coûts des examens, des médicaments et des consultations/hospitalisations

Examens	Coût sans assurance (FCFA*)	Coût avec assurance (FCFA*)
Hématologie	40.000	8.000
Biochimie Sérologie	89.375 43.750	17.875 8.750
Radiologie	50.000	26.400
Total	223.125	51.025
Médicaments	20.810	4.432
Forfait d´hospitalisation (hospitalisation + médicaments + examens)	387.000	77.400
Forfait première consultation (examens + consultation)	258.875	51.775
Forfait suivi régulier (examens + consultation)	37.000	7400

*Taux de change 1 Franc CFA= 0,0015 Euro

**Tableau 4:** répartition des patients selon la gestion quotidienne de la maladie

	Effectif	Fréquence (%)
**Obstacle aux activités de la vie quotidienne**		
Oui	29	33,4
A peu près	7	8,0
Non	51	58,6
Total	87	100,0
**Obstacle à la stabilité professionnelle**		
Oui	9	20,5
A peu près	1	2,6
Non	41	76,9
**Total**	51	100,0
**Support pour surmonter la maladie au quotidien (N=88)**		
Famille/Amis	46	52,5
Foi/Prière	27	30,7
Acceptation de la maladie	31	35,2
Rareté des crises	2	2,3
**Sentiment d´être compris**		
Oui	67	76,1
A peu près	4	4,5
Non	17	19,3
Total	88	100,0

### Gestion de la maladie (coût humain)

Pour 29 (33,7%) patients, la drépanocytose était un obstacle aux activités de la vie quotidienne alors que pour 9 (20,5%) elle constituait un obstacle à la stabilité professionnelle. Le sentiment d´être compris au quotidien était retrouvé chez 67 (76,1%) drépanocytaires. La majorité des patients (46) soit 52,5% trouvaient un soutien affectif dans leur famille et chez leurs amis. La religion et l´auto-motivation étaient une source de soutien dans 30,7% et 26,1% des cas respectivement (Tableau 4).

**Connaissance de la maladie:** un total de 62 (70,5%) patients disait connaitre la drépanocytose. Leur source d´information sur les aspects cliniques et évolutifs de la maladie était dans la grande majorité des cas l´équipe médicale 75 (82,2%).

## Discussion

Cette étude a permis d´évaluer la drépanocytose homozygote en consultation d´hématologie au CHUL avec 233 dossiers dont 145 inexploitables. Ces valeurs sont du même ordre de grandeur que celles observées par Kueviakoe MDI *et al*. [6]. L´absence d´exploitation de 145 dossiers, en l´absence de registre, permet de penser que ce nombre est sous-estimé. Notre étude a montré que la drépanocytose adulte affecte la tranche d´âge comprise entre 25 et 29 ans, supérieure à celle observée en Côte d´Ivoire et au Sénégal [7, 8]. L´existence dans ces pays d´unités de prise en charge de l´adulte drépanocytaire permettant une transition organisée de la consultation pédiatrique à celle de l´adulte drépanocytaire, pourrait être un argument expliquant cet écart. Cet âge relativement élevé démontre que la drépanocytose n´est plus une maladie de l´enfance. Les femmes étaient plus nombreuses que les hommes. Cette prédominance féminine est également retrouvée par Dokekias *et al*. et Simo Louokdom [9, 10]. Elle rend compte du profil des populations dans ces pays où les femmes sont les plus nombreuses. Quatre-vingt pourcents des patients de l´étude étaient célibataires, ce taux est supérieur à celui noté par Matthie *et al*. [11]. La stigmatisation dont ils sont victimes, les hospitalisations répétées et la peur de la transmission génétique de la maladie pourrait expliquer ce pourcentage.

En plus des difficultés à tisser des relations durables, la drépanocytose, et plus précisément ses complications, entrainent un absentéisme scolaire. Les cycles primaires et secondaires sont souvent perturbés retardant l´accès à l´université. Quarante-deux pourcents des patients ont pu faire des études supérieures. Ce chiffre est inférieur aux 61% retrouvés par Tchicaya *et al*. [12] alors que dans l´étude réalisée par Dokekias *et al*. [9], 50% des patients atteignaient le niveau Brevet du Premier Cycle (BEPC). De façon générale, ce niveau d´instruction permet au drépanocytaire d´avoir une meilleure connaissance de la maladie. Cependant, malgré un niveau d´études supérieures relativement satisfaisant, 59,1% des patients étaient sans emploi. Ce qui est proche des 69,84% rapportés par Elira Dokekias A *et al*. [9].

La presque totalité des patients avaient une assurance maladie couvrant les frais de santé. Les patients qui disposaient d´un revenu mensuel représentaient 40,9% de la population d´étude et 58,3% d´entre eux avaient un revenu mensuel avoisinant les 200.000FCFA (305 euros) pour un SMIG de 80.000FCFA (122 euros) au Gabon. Ngolet *et al*. au Congo retrouvaient des taux similaires [13]. L´accès à cette assurance maladie facilitait la prise en charge des non-salariés. En l´absence de prise en charge à 100%, les tickets modérateurs, estimés à 20% du coût total des prestations de soins, étaient réglés par la famille et/ou le revenu mensuel des drépanocytaires salariés qui le pouvaient. Le coût total des examens régulièrement réalisés par les patients était de 223 125 F CFA sans assurance et 51 025F CFA avec assurance. Les détails de ce dernier montant sont proches de ceux retrouvés au Burkina Faso par Ouédraogo SO *et al*. sur une étude réalisée chez les enfants [14]. Les frais d´hospitalisation avec assurance étaient proches de l´étude de Ngolet *et al*. qui retrouvaient un coût global moyen de 103.110 FCFA pour les examens et le traitement [13].

Il faut cependant souligner que l´évaluation médico-économique des drépanocytaires SS adultes reste difficile et nécessite une étude de paramètres complexes [15]. Notre étude n´a pu évaluer que des coûts directs induits par la prise en charge des patients, rarement rapportés dans d´autres études. Le coût humain ne semblait pas au premier plan. Seuls 29 (33,4%) patients ont reconnu que la maladie représentait une gêne dans la réalisation des activités de la vie quotidienne et pour 9 (20,5%), elle constituait un obstacle à la stabilité de l´emploi. La rareté de la survenue des crises chez ces adultes justifiait le fait que pour plus de la moitié d´entre eux, la maladie ne représentait pas un obstacle aux activités quotidiennes ou professionnelles. Cette relative stabilité pourrait être expliquée par un accompagnement familial permanent. La moitié des patients se sentaient compris et soutenus par leur entourage (famille et amis) grâce à leur soutien affectif. Les autres puisaient leur force dans la religion et l´auto-motivation. Ces différentes sources de motivations étaient également retrouvées par Matthie *et al*. chez des drépanocytaires afro-américains [11]. L´absence d´une structure de suivi de l´adulte les contraint en pédiatrie où ils sont vus depuis leur enfance. De ce fait, peu de données existent sur la drépanocytose de l´adulte dans le pays. Une enquête comparative du vécu et des coûts entre les patients vus en consultation adultes et ceux demeurés en pédiatrie aurait permis d´obtenir des arguments forts pour établir un plaidoyer pour l´ouverture d´une structure dédiée au suivi de la drépanocytose.

## Conclusion

Cette étude a permis de montrer que les patients drépanocytaires qui adhèrent au suivi en consultation adulte sont bien intégrés dans la société. Ils ont pour la plupart un niveau d´instruction qui leur permet de comprendre leur maladie et une assurance santé qui constitue une aide pour leur prise en charge. Cette dernière est cependant rendue difficile par le manque d´une unité qui leur est dédiée pour le suivi en cas de complication aiguë. La réalisation d´une étude qui tiendrait compte des patients non réguliers, et dont les données seraient comparées à notre population d´étude est un préalable non négligeable pour la conception et l´organisation de cette unité.

### Etat des connaissances sur le sujet

- La drépanocytose est la maladie génétique la plus répandue;
- La transition de la consultation pédiatrique à celle de l´adulte est au cœur de la prise en charge des adultes car si elle est mal organisée, elle compromet gravement la survie du patient.

### Contribution de notre étude à la connaissance

- Déterminer un profil de patients adultes susceptibles de réussir leur transition et de venir régulièrement à la consultation;
- Le praticien pourra se servir des caractéristiques de la population décrite dans l´étude pour établir un contrat de confiance avec le consultant.

## Remerciements

Nous remercions M. Emmanuel E qui a permis l´analyse statistique des données.
